# Killer immunoglobulin‐like receptor genotypes and chronic myeloid leukemia outcomes after imatinib cessation for treatment‐free remission

**DOI:** 10.1002/cam4.2371

**Published:** 2019-07-09

**Authors:** Pierre‐Yves Dumas, Emilie Bérard, Claire Bréal, Stéphanie Dulucq, Delphine Réa, Franck Nicolini, Edouard Forcade, Melody Dufossée, Jean‐Max Pasquet, Béatrice Turcq, Audrey Bidet, Noel Milpied, Julie Déchanet‐Merville, Xavier Lafarge, Gabriel Etienne, François‐Xavier Mahon, French Intergroup in Chronic Myeloid Leukemia

**Affiliations:** ^1^ Service d'Hématologie Clinique et Thérapie Cellulaire CHU Bordeaux F-33000 Bordeaux France; ^2^ Institut National de la Santé et de la Recherche Médicale INSERM U1035 Bordeaux France; ^3^ Service d'Epidémiologie Centre Hospitalier Universitaire de Toulouse Toulouse France; ^4^ UMR 1027 INSERM‐Université de Toulouse III Toulouse France; ^5^ Laboratoire d'Hématologie CHU Bordeaux F-33000 Bordeaux France; ^6^ Service d'Hématologie Hôpital Saint Louis Paris France; ^7^ Service d'Hématologie and INSERM U590 CRCL, Centre Léon Bérard Lyon France; ^8^ Institut National de la Santé et de la Recherche Médicale INSERM U1218 Bordeaux France; ^9^ Centre National de la Recherche Scientifique, ImmunoConcEpT, UMR 5164 Bordeaux France; ^10^ Laboratoire d'Immunogénétique Etablissement Français du Sang Bordeaux France; ^11^ Centre de Lutte contre le Cancer Institute Bergonié Bordeaux France

**Keywords:** chronic myeloid leukemia, imatinib, killer immunoglobulin‐like receptors, natural killer, treatment‐free remission

## Abstract

**Background**

Natural Killer (NK) cells are innate lymphoid cells that can be cytotoxic toward a large panel of solid tumors and hematological malignancies including chronic myeloid leukemia (CML). Such a cytotoxicity depends on various receptors. Killer immunoglobulin‐like receptors (KIR) belong to these receptors and are involved in maturation process, then in the activation abilities of NK cells. Methods: We investigated the prognostic impact of the *KIR2DL5B* genotype in 240 CML patients included in two clinical trials investigating tyrosine kinase inhibitors (TKI) discontinuation: STIM and STIM2. Results: After adjustment for standard risk factors in CML, we found that the inhibitory receptor *KIR2DL5B‐*positive genotype was independently related to a delayed second deep molecular remission (HR 0.54, 95% CI [0.32‐0.91], *P = *0.02) after TKI rechallenge but not to time to first deep molecular remission or treatment‐free remission rates. Conclusion: These results suggest that *KIR2DL5B* could carry a role in lymphocyte‐mediated control of leukemic residual disease control in patient with CML relapse.

## 1 INTRODUCTION

Chronic myeloid leukemia (CML) is a clonal myeloproliferative disorder caused by the constitutively active chimeric *BCR‐ABL1* tyrosine kinase. CML patients have benefited from tyrosine kinase inhibitors (TKI) and prognosis has dramatically changed in the two last decades. Nevertheless, some new issues should now be challenged: quality of life and economic impact of treating patients during whole lifetimes since TKI‐treated CML patients have a near‐normal life expectancy.^1^ Recently, several trials demonstrated that TKI could be stopped in patients with a sustained deep molecular response (DMR), leading to the concept of treatment‐free remission (TFR).^2^, ^3^ However, for half of the patients, TKI must be reinitiated. Therefore, biomarkers are needed to better predict TFR, to keep on improving the results of stopping treatment strategies.^4^

Natural killer (NK) cells are innate lymphoid cells and critical components of the immune system, providing a front‐line defense against tumor cells and supporting the initiation of the adaptive immune reaction. NK cells are subdivided in two subsets: CD56^dim^ that exerts the cytotoxic effect and CD56^bright^ that produces the immunoregulatory cytokines, such as IFN‐γ and TNF. NK cell functions are under the control of surface receptors, most of which belong to four families: killer immunoglobulin‐like receptors (KIR), leukocyte immunoglobulin‐like receptors, natural cytotoxicity receptors, and C‐type lectin receptors. NK cell dysfunction is a well‐known mechanism of immune escape worsening with CML progression.^5^ Various mechanisms are involved: shedding of ligands by leukemic cells,^6^ downregulation of activating receptors,^7^ expansion of myeloid‐derived suppressor cells that promotes the recruitment of T_Reg_ and impairs NK cells in a membrane‐bound TGFβ1 manner.^8^ The role of NK cells in a sustained TFR has been documented in several studies: a high number of NK cells at the time of TKI discontinuation is predictive of better disease control,^9^ and more precisely mature cytotoxic CD56^dim^ subset, that is an independent prognostic factor for TFR.^10^ However, NK cell‐related mechanisms specifically involved in disease control for half of the patients who successfully discontinue TKI are still poorly understood.

The receptor KIR2DL5 belongs to KIR receptor family and possesses a unique combination of genetic, structural, and functional hallmarks that confer an inhibitory function when binding to its unknown ligand.^11^ It is encoded by two genes, *KIR2DL5A* and *KIR2DL5B*, present in half of the Caucasian population.^12^ The expression of KIR2DL5 has solely been described in CD8^+^ T cells and CD56^dim^ NK subset.^13^ In the setting of CML, *KIR2DL5A* and *KIR2DL5B* genotypes have been associated with a decrease in the rate of 12‐month molecular response 14 and 2‐year complete cytogenetic response.^15^ Recently, the specific *KIR2DL5B* genotype has been shown to predict a bad prognosis through various outcomes in CML in a first‐line imatinib strategy, including transformation‐free survival, suggesting an important role in CML immune escape.^16^

In the current study, we investigated the role of *KIR2DL5B* genotype with the objective to find a biomarker to better predict outcomes in a stopping imatinib strategy for CML.

## 2 MATERIALS AND METHODS

### 2.1 Patients

Between July 2007 and December 2009, 100 patients ≥18 years with CML, were included in the French multicentric STIM trial (NCT00478985). Patients who received IFN‐α prior to imatinib have been excluded from this study (N = 50) because of potential confounding effect in NK cell‐mediated response. From which, we have genotyped all the samples (100%) for *KIR*. Between April 2011 and March 2015, 218 patients ≥18 years with CML, were included in the French multicentric STIM2 trial (NCT01343173). From which, we have genotyped 193 samples (88.5%) for *KIR*. These trials included both patients with chronic or accelerated phase CML, treated only with imatinib as a single agent for at least 3 years, allowing a DMR, defined as no detectable *BCR‐ABL1* transcript with at least a sensitivity >4.5 logs according to the European Leukemia Net recommendations for minimal residual disease evaluation. The DMR must be sustained for two consecutive years and on a minimum of five data points before stopping imatinib. Patients with previous allogenic stem cell transplantation were excluded. After imatinib cessation, minimal residual disease monitoring was performed in laboratories according to European Leukemia Net recommendations, monthly for the first 12 months, then every 2 months in year 2, and every 3 months thereafter. Molecular recurrence triggering reinitiation of TKI was defined according to *BCR‐ABL1* ^IS^ transcript level: in case of loss of major molecular response (MMR) on a single assessment or if two consecutive assessments showed detectable transcripts with ≥1‐log increase between measurements. Written informed consent was obtained from all patients and the protocols were approved by the ethics committees.^2^, ^17^

### 2.2 KIR genotyping and haplotype group assignment

*KIR* genotype has been performed in samples prospectively collected and frozen with the Lifecodes KIR‐SSO typing KIT for use with Luminex^®^ (Immucor Transplant diagnostics, Inc, Stamford, USA) according to manufacturer instructions. This first genotyping strategy allowed us to determine status for *KIR2DL1*, *KIR2DL2*, *KIR2DL3*, *KIR2DL4*, *KIR2DL5*, *KIR3DL1*, *KIR3DL2*, *KIR3DL3*, *KIR2DS1*, *KIR2DS2*, *KIR2DS3*, *KIR2DS4*, *KIR2DS5*, and *KIR3DS1*. For patients who were *KIR2DL5‐*positive, we used the Olerup SSP KIR Genotyping^®^ kit (Olerup SSP AB, Stockholm, Sweden) according to the manufacturer instructions, to distinguish *KIR2DL5A* and/or *KIR2DL5B‐*positive patients, also allowing to control the results obtained with Lifecodes KIR‐SSO typing KIT. Patients were also assigned to the *B/x* or *A/A* genotype as previously described.^18^ Briefly, detection of at least one of the KIR *B* haplotype‐defining loci dictated that the genotype contains at least one *B* haplotype and such samples were assigned to the genotype *B/x*. Samples lacking all KIR *B* haplotype‐defining loci were assigned to the genotype *A/A*.

### 2.3 Statistical methods

#### 2.3.1 Endpoints

The primary endpoint was TFR, prospectively measured from the date of imatinib discontinuation to the date of the earliest of the following: loss of MMR, restart of imatinib treatment, increase ≥1‐log *BCR‐ABL1*, progression to blastic crisis or death from any cause. Molecular events were detected according to European Leukemia Net recommendations, monthly for the first 12 months, then every 2 months in year 2, and every 3 months thereafter. The secondary endpoints were time to first DMR and time to second DMR. Time to first DMR was retrospectively measured from the date of the initiation of imatinib to the date of the first DMR. Time to second DMR was prospectively measured from the date of TKI treatment restart to the date of the second DMR.

#### 2.3.2 Sample size

Before doing any analyses, we assessed the power of the study for the primary endpoint: 129 relapses or deaths provided a power greater than 90% to detect a Hazard Ratio (HR) for TFR ≥1.80 (for *KIR2DL5B‐*positive vs* KIR2DL5B‐*negative groups) with a two‐sided type‐1 error rate of 5% (alpha = 0.05) for the comparison of two exponential survival distributions.^19^ Moreover, 129 relapses or deaths provided the power for adjusted analysis incorporating up to 12 factors.^20^

### 2.4 Statistical analysis

Statistical analysis was performed on STATA statistical software, release 14.2 (STATA Corporation, College Station, TX, USA). Univariate survival analyses assessed hazard ratio (HR) and 95% confidence intervals (95% CI) using a standard Cox model and Kaplan‐Meier survival curves. Multivariate analysis initially included *KIR2DL5B* together with potential confounding factors (associated with endpoint in univariate analysis with a *P* < 0.20): others factors include *KIR*, age, gender, Sokal risk score (for TFR, time to first and second DMR) and imatinib therapy duration, time to first DMR, DMR duration before discontinuation of imatinib (for TFR and time to second DMR). A backward analysis was then applied until only variables significantly and independently associated with TFR, time to first or second DMR (*P* < 0.05) remained. The proportional‐hazard assumption was tested for each covariate of the Cox model using the “log‐log” plot method curves and was always met. When the linearity hypothesis was not respected, continuous variables were transformed into ordered data using quartiles. Interactions between independent covariates and *KIR2DL5B* were tested (none were significant). All reported *P*‐values were two‐sided, and the significant threshold was <0.05.

## 3 RESULTS

### 3.1 Patients' characteristics, time to first DMR and *KIR2DL5B* genotype

As presented in study flowchart (Figure S1), 49 of the 100 patients enrolled in the STIM trial were included in the current study. Fifty patients have been excluded because of previous treatment by IFN‐α. One patient has been excluded because of *KIR* genotyping failure. Among 218 patients included in the STIM2 trial, 193 were initially included in the current study but two patients were in *KIR* genotyping failure leading to a final sample of 191 patients. Baseline characteristics of the 240 patients included are detailed in Table 1. Briefly, 52.5% were female and the Sokal score at diagnosis was low, intermediate, and high in 44.8%, 41.8% and 13.4%, respectively. Single‐agent imatinib was given as the first‐line TKI therapy in all patients for a median duration of about 6 years (range 2.9‐12.5) before TKI discontinuation. The median age at discontinuation of imatinib was 60.8 years (range 24.2‐91.1) and the median follow‐up after imatinib discontinuation for patients who did not relapse was 24.1 months (range 6.0‐92.5). Median time to first DMR was 25.8 months (range 2.7‐104.6) and median of first DMR duration was 3.1 years (range 1.9‐10.4). The KIR gene frequencies are described in Table 2. Sixty (25%) patients were *KIR2DL5B‐*positive, half were also *KIR2DL5A‐*positive,^21^ and we found a strong positive linkage disequilibrium between *KIR2DL5B* and *KIR2DL2*, *KIR2DS2*, and* KIR2DS3,* as previously reported.^21^, ^22^

**Table 1:** Baseline characteristics of 240 chronic myeloid leukemia patients

	Total N = 240 (100%)	*KIR2DL5B* negative N = 180 (75%)	*KIR2DL5B* positive N = 60 (25%)	*P*‐value
Clinical trial‐N (%)				
STIM1	49 (20.4)	39 (21.7)	10 (16.7)	0.41
STIM2	191 (79.6)	141 (78.3)	50 (83.3)	
Age at discontinuation of imatinib (years)				
Median (IQR)	60.8 (51.4‐70.7)	60.6 (51.1‐69.1)	64.1 (51.5‐72.7)	0.71
Range	24.2;91.1	24.2;91.1	27.0;83.9	
Gender‐N (%)				
Male	114 (47.5)	82 (45.6)	32 (53.3)	0.30
Female	126 (52.5)	98 (54.4)	28 (46.7)	
Sokal risk score at diagnosis‐N (%)				
Low	107 (44.8)	83 (46.1)	24 (40.7)	0.75
Intermediate	100 (41.8)	73 (40.6)	27 (45.8)	
High	32 (13.4)	24 (13.3)	8 (13.6)	
Imatinib duration (years)				
Median (IQR)	6.0 (4.6‐7.9)	6.0 (4.6‐8.0)	5.8 (4.4‐7.4)	0.28
Range	2.9;12.5	2.9;12.0	3.2;12.5	
Time to first DMR (months)				
Median (IQR)	25.8 (16.5‐46.8)	25.9 (17.2‐47.4)	25.0 (15.4‐42.4)	0.77
Range	2.7;104.6	2.7;104.6	5.5;83.0	
First DMR duration (years)^a^				
Median (IQR)	3.1 (2.4‐4.4)	3.1 (2.4‐4.3)	3.1 (2.4‐4.5)	0.93
Range	1.9;10.4	2.0;10.4	1.9;9.7	
Molecular recurrence‐N (%)^b^	128 (53.3)	100 (55.6)	28 (46.7)	0.23
Deaths‐N (%)	3 (1.3)	2 (1.1)	1 (1.7)	1.00

Abbreviations: DMR, deep molecular remission; KIR, killer immunoglobulin‐like receptors; IQR, interquartile range.

a Before discontinuation of imatinib.

b After discontinuation of imatinib.

**Table 2:** KIR positive genotype of 240 chronic myeloid leukemia patients‐N (%)

	Total N (%)		*KIR2DL5B* negative‐N (%)		*KIR2DL5B* positive‐N (%)		*P*‐value
	240	(100)	180	(100)	60	(100)	
*KIR2DL1*	231	(96.3)	172	(95.6)	59	(98.3)	0.46
*KIR2DL2*	121	(50.6)	62	(34.6)	59	(98.3)	<0.01
*KIR2DL3*	218	(90.8)	174	(96.7)	44	(73.3)	<0.01
*KIR2DL4*	240	(100)	180	(100)	60	(100)	–
*KIR2DL5A*	91	(37.9)	61	(33.9)	30	(50.0)	0.03
*KIR3DL1*	220	(91.7)	171	(95.0)	49	(81.7)	<0.01
*KIR3DL2*	240	(100)	180	(100)	60	(100)	–
*KIR3DL3*	240	(100)	180	(100)	60	(100)	–
*KIR2DS1*	91	(37.9)	61	(33.9)	30	(50.0)	0.03
*KIR2DS2*	121	(50.4)	63	(35.0)	58	(96.7)	<0.01
*KIR2DS3*	66	(27.5)	13	(7.2)	53	(88.3)	<0.01
*KIR2DS4*	222	(92.5)	173	(96.1)	49	(81.7)	<0.01
*KIR2DS5*	77	(32.1)	53	(29.4)	24	(40.0)	0.13
*KIR3DS1*	97	(40.4)	63	(35.0)	34	(56.7)	<0.01

Abbreviation: KIR, killer immunoglobulin‐like receptors.

**Figure 1:**
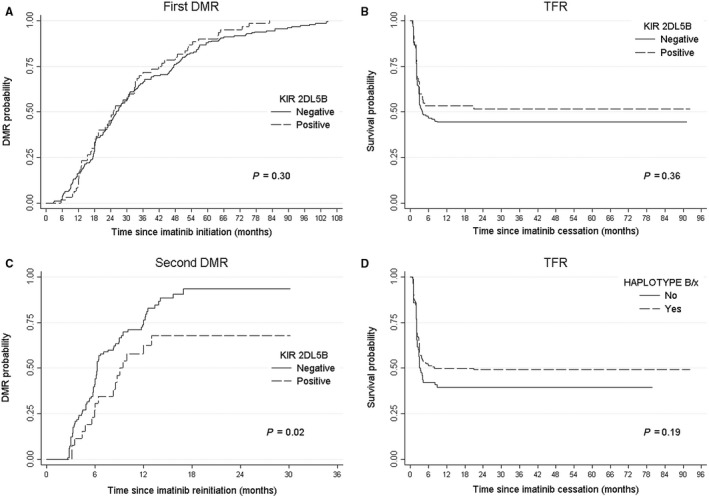
Outcomes in CML patients according to KIR genotype. A, First DMR, which did not differ significantly according to *KIR2DL5B* status, B, TFR, which did not differ significantly according to *KIR2DL5B* status, C, Second DMR, which differed significantly according to *KIR2DL5B* status. D, TFR, which did not differ significantly according to haplotype assignment; CML, Chronic Myeloid Leukemia; DMR, Deep molecular response; KIR, Killer Immunoglobulin‐like Receptors; TFR, Treatment‐free remission.

No difference was highlighted in patients' characteristics or relapse incidence depending on *KIR2DL5B* status nor any other *KIR* genotype status (Table 1 and Table S1). Part of STIM patients were also included in the recently published IMMUNOSTIM study,^10^ so we analyzed NK cell levels in patients included in this ancillary study for which we disposed *KIR* genotype data: at the time of imatinib discontinuation, *KIR2DL5B‐*negative and positive patients had similar levels of NK cells, CD56^bright^ subset, and cytotoxic CD56^dim^ subset (Figure S2). Time to first DMR was not influenced by *KIR2DL5B* status nor any other *KIR* genotype (Figure 1A and Table S2).

### 3.2 Treatment‐free remission according to *KIR2DL5B* status and relapsing patients' characteristics

**Figure 2:**
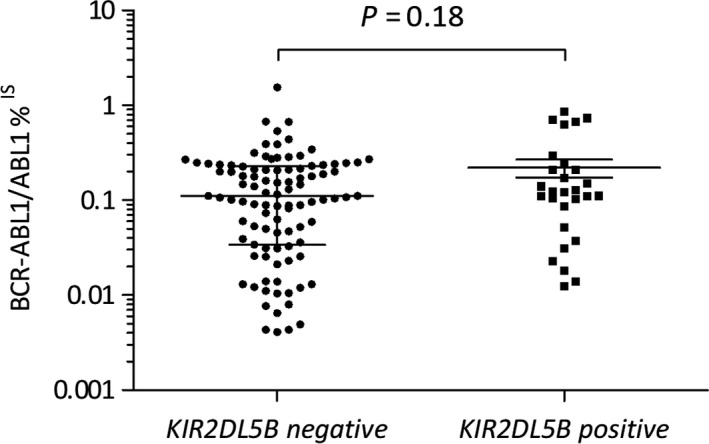
*BCR‐ABL1/ABL1* ^IS^ and *KIR2DL5* expression levels according to *KIR2DL5* genotype. Scatter dot plots represent *BCR‐ABL1*%^IS^ for each individual relapsing patient at relapse confirmation, median values and interquartile range are also shown (N = 128)

At the time of analysis, 128 (53.3%) patients experienced a molecular recurrence according to protocol‐defined criteria after a median delay of 2.1 months (range 0.7‐8.9) following imatinib discontinuation (Table 3). The median *BCR‐ABL1/ABL1* ^IS^ ratio at the time of molecular recurrence was 0.1105 (range 0.0041‐1.546) and 0.1218 (range 0.0124‐0.8541), for negative and positive *KIR2DL5B* patients (*P = *0.18), respectively (Figure 2).

**Table 3:** Characteristics of 128 chronic myeloid leukemia patients in recurrence after TKI discontinuation

	Total N (%) 128 (100%)		*KIR2DL5B* negative N (%) 100 (78.1%)		*KIR2DL5B* positive N (%) 28 (21.9%)		*P*‐value
Time to recurrence (months)^a^							
Median (IQR)	2.1 (1.5‐3.0)		2.1 (1.6‐3.0)		2.0 (1.3‐2.9)		0.82
Range	0.7;8.9		0.8;8.9		0.7;4.8		
Treatment at recurrence^a^							
n (%)	125	(97.7)	97	(97.7)	28	(100)	1.00
Imatinib	116	(92.8)	90	(92.8)	26	(92.9)	0.41
Dasatinib	4	(3.2)	4	(4.1)	0	(0)	
Nilotinib	4	(3.2)	2	(2.1)	2	(7.1)	
Bosutinib	1	(0.8)	1	(1.0)	0	(0)	
Second DMR^b^							
Yes	97	(77.6)	80	(82.5)	17	(60.7)	0.01
No	28	(22.4)	17	(17.5)	11	(39.3)	
Time to second DMR (months)							
Median survival (IQR)	6.4 (4.8‐12.3)		6.2 (4.3‐12.0)		9.0 (5.9‐NR)		0.03

Abbreviations: DMR, deep molecular remission; KIR, killer immunoglobulin‐like receptors; IQR, interquartile range; NR, not reached.

a After imatinib discontinuation with a median follow‐up of 24.1 months (IQR 23.5‐26.0, range 6.0;92.5) for patients who did not relapse without any statistical difference depending on *KIR2DL5B* status (*P* = 0.78).

b For relapsed patients who rechallenged TKI treatment at recurrence (N = 125).

Univariate analyses identified potential confounding factors associated with TFR in univariate analysis with *P* < 0.20 (Table S3) such as high‐risk Sokal score (HR 1.56, 95% CI [0.95‐2.56], *P = *0.08), imatinib duration >6 years (HR 0.66, 95% CI [0.46‐0.93], *P = *0.02), time to first DMR ≥ 26 months (HR 0.77, 95% CI [0.54‐1.09], *P = *0.14), DMR duration before imatinib discontinuation >4.4 years (HR 0.58, 95% CI [0.37‐0.91], *P = *0.02), *KIR2DL2* (HR 0.79, 95% CI [0.56‐1.12], *P = *0.19) and *KIR2DS3* (HR 0.76, 95% CI [0.51‐1.14], *P = *0.19). In multivariate analysis, DMR duration before imatinib discontinuation > 4.4 years (aHR 0.58, 95% CI [0.37‐0.91], *P = *0.02) was associated with TFR while *KIR2DL5B‐*positive genotype was not (aHR 0.84, 95% CI [0.55‐1.27], *P = *0.40) (Table S3). The median TFR was 4.0 months (Inter Quartile Range (IQR) 2.04‐not reached) for *KIR2DL5B‐*negative patients and was not reached (IQR 2.07‐not reached) for *KIR2DL5B‐*positive patients (*P = *0.36, Figure 1B).

Molecular recurrences were rechallenged by imatinib in 116 (92.8%) patients treated at relapse (N = 125), nine patients received a second generation TKI. Among these relapses, 124 (96.9%) occurred within the first 6 months after stopping imatinib. The four remaining patients displayed later relapses, between 7 and 9 months, none of them were *KIR2DL5B* positive.

### 3.3 Role of *KIR* genotype in time to second deep molecular remission

*KIR* genotype failed to predict time to first DMR and TFR, we were finally interested in its ability to predict time to second DMR for the 125 patients who experienced recurrence after imatinib cessation and rechallenged TKI. At the time of analysis, 77.6% of patients reached a second DMR: 80 (82.5%) and 17 (60.7%) (*P = *0.01) for *KIR2DL5B‐*negative and positive patients (Table 3), respectively. Median time to second DMR was 6.2 (IQR 4.3‐12.0) vs 9.0 (IQR 5.9‐not reached) months (*P = *0.03) for *KIR2DL5B‐*negative and positive patients, respectively (Table 3). In univariate analysis, factors significantly associated with time to second DMR are described in Table 4. In multivariate analysis, time to first DMR ≥ 26 months (HR 0.58, 95% CI [0.38‐0.88], *P = *0.01) and *KIR2DL5B‐*positive status (HR 0.54, 95% CI [0.32‐0.91], *P = *0.02) were associated with a longer delay before second DMR (Figure 1C) while treatments at recurrence were similar in both groups (Table 3) with a median follow‐up from relapse to last follow‐up examination for relapsing patients about 14 months (IQR 10‐15).

**Table 4:** Cox model for factors associated with time to second DMR among 125 relapsed patients treated at recurrence

	N	Univariate analysis			Multivariate analysis		
		HR	95% CI	*P*‐value	aHR	95% CI	*P*‐value
Age > 51 years^a^	96	0.86	0.55‐1.36	0.53	–	–	–
Gender female	62	1.21	0.81‐1.81	0.35	–	–	–
Sokal^b^							
Intermediate	50	0.86	0.55‐1.33	0.49	–	–	–
High	22	0.73	0.41‐1.31	0.29	–	–	–
Imatinib > 6 years^c^	50	0.54	0.36‐0.83	<0.01	–	–	–
Time to first DMR ≥ 26 months^c^	55	0.60	0.40‐0.91	0.02	0.58	0.38‐0.88	0.01
DMR duration > 4.4 years^d^	20	0.59	0.34‐1.05	0.07	–	–	–
*KIR2DL1*	121	0.76	0.24‐2.40	0.64	–	–	–
*KIR2DL2*	57	0.94	0.63‐1.41	0.78	–	–	–
*KIR2DL3*	116	2.08	0.85‐5.13	0.11	–	–	–
*KIR2DL4*	125	–	–	–	–	–	–
*KIR2DL5*	61	0.59	0.39‐0.89	0.01	–	–	–
*KIR2DL5A*	48	0.79	0.52‐1.20	0.27	–	–	–
*KIR2DL5B*	28	0.56	0.33‐0.95	0.03	0.54	0.32‐0.91	0.02
*KIR3DL1*	117	2.02	0.74‐5.52	0.17	–	–	–
*KIR3DL2*	125	–	–	–	–	–	–
*KIR3DL3*	125	–	–	–	–	–	–
*KIR2DS1*	48	0.77	0.50‐1.17	0.22	–	–	–
*KIR2DS2*	58	0.95	0.64‐1.42	0.81	–	–	–
*KIR2DS3*	30	0.74	0.45‐1.21	0.23	–	–	–
*KIR2DS4*	118	2.48	0.78‐7.86	0.12	–	–	–
*KIR2DS5*	39	0.74	0.48‐1.16	0.19	–	–	–
*KIR3DS1*	49	0.77	0.51‐1.17	0.23	–	–	–

Abbreviations: aHR, adjusted hazard ratio; CI, confidence interval; DMR, deep molecular response; HR, hazard ratio.

a First quartile.

b Reference is low Sokal (N = 53).

c Median.

d Fourth quartile.

### 3.4 Role of *A/A* and *B/x* haplotypes in CML

Since *KIR2DL5* genes allow for haplotype assignment, we studied this model in the current cohort. From the genotype, we determined whether each patient was of *A/A* or *B/x* haplotype. For the *B/x* patients, we further determined whether their *B* haplotype genes were in the centromeric or telomeric part of the KIR locus to calculate the KIR *B*‐content score which gives the total number of centromeric and telomeric motifs containing *B* haplotype genes (Table S4). Frequencies observed in this cohort were similar to that observed in previous studies for healthy donors.^18^ The Kaplan‐Meier estimates of the median time to first DMR was 25.9 months (IQR 17.4‐46.5) and 25.1 months (IQR 16.1‐47.4) for *A/A* and *B/x* patients (*P = *0.74), respectively. The Kaplan‐Meier estimates of the median time to second DMR was 6.4 months (IQR 4.8‐9.5) and 6.4 months (IQR 4.4‐13.4) for *A/A* and *B/x* patients (*P = *0.12), respectively. Finally, the Kaplan‐Meier estimates of the median TFR was 3.0 months (IQR 2.0‐not reached) and 8.0 months (IQR 2.1‐not reached) for *A/A* and *B/x* patients (*P = *0.19), respectively (Figure 1D).

## 4 DISCUSSION

This study investigated, in a large cohort of CML patients, *KIR* genotype prognostic effects with a focus on *KIR2DL5B* for stopping imatinib strategy. *KIR2DL5B* genotype was associated with the achievement of a second DMR after treatment reinitiation but not the achievement of first DMR and TFR. Clinical impact of such a small difference in median time to second DMR is debatable but probability to obtain a second DMR remains relevant, particularly in light of previous study about *KIR2DL5B* in time to obtain a first DMR.^16^

The role of NK cells is well known in disease control of myeloid malignancies, particularly in the setting of KIR‐HLA mismatch, as previously published in haploidentical allogenic stem cell transplantations by Velardi's group.^23^ The potential of KIR appears to be at least equally important in an autologous setting. Indeed, everyone has its own HLA and KIR systems encoded by genes located on chromosomes 6 and 19, respectively. Thus, HLA‐KIR mismatch exists in everyone and some of our lymphocytes do not have KIR that recognizes our own HLA system. Prognostic role of such mismatches in an autologous setting have recently been highlighted in acute myeloid leukemia.^24^

Marin et al showed in 2012 that *KIR2DS1* was the only independent factor for lower probability of achieving complete cytogenetic response, lower progression‐free survival and overall survival in CML.^15^ Recently, Yeung et al found that *KIR2DL5B* was associated with a lower event‐free survival and was the only independent factor associated with inferior MMR in a response‐directed sequential imatinib/nilotinib strategy. *KIR2DS1* was not associated with outcomes in this second study.^16^ The current one, based on a large cohort of two prospective trials, does not reproduce these results in front‐line efficacy of TKI, neither for *KIR2DS1* nor for *KIR2DL5B*, but the population of CML patients was not similar. Indeed, patients enrolled in the STIM and STIM2 trials were older and selected on their ability to reach a DMR for at least 2 years. Moreover, this part of the study is based on retrospective data and no over‐interpretation of results in time to first DMR should be made.

Increased proportions of mature NK cells have recently been shown by Ilander et al to be associated with successful imatinib discontinuation in EURO‐SKI trial. Part of these patients (N = 43) have been KIR‐genotyped and classified according to haplotype *B/x* and *A/A* subtypes.^9^ Group *A* haplotype has a fixed number of genes that encode mostly inhibitory receptors with the exception of *2DS4*, whereas group *B* haplotypes have variable gene content including additional activating receptor genes. Donor *B/x* haplotype has been associated with survival benefit for patients undergoing unrelated allogenic stem cell transplantations for AML.^18^, ^25^ Among 43 patients, Ilander et al did not find any haplotype effect in an autologous setting for CML, whereas Caocci et al found an effect among a cohort of 36 patients.^26^ In the current study among a large cohort of 240 patients, we found a trend in better TFR for haplotype *B/x* patients, those carriers of more activating KIR receptor‐gene, able to trigger NK cells' cytotoxicity.

Role of NK cells and other innate lymphoid cells in cancer has been recently reviewed.^27^ Various mechanisms could be involved in the role of NK cells in CML such as ligands shedding in microenvironment, downregulation of activating receptors such as NKG2D and NCRs, clonal expansion of MDSC.^28^ Moreover, several studies support that TKI exert an immunomodulatory off‐target effect on immune system,^29^ with a direct effect on NK cell number, an increase of previously described downregulated receptors, and an inhibition of T_Reg_ immunosuppressive function and MDSC expansion.

Finally, we can argue that the role of NK cells in CML control seems important after imatinib discontinuation,^9^, ^10^, ^30^ but currently, precise mechanisms associated with disease control are still unclear and *KIR* genotype, as reflects of NK cell ability to exert a cytotoxicity against tumor cells is only partial.^7^

## CONFLICT OF INTEREST

The authors declare no potential conflict of interest.

## AUTHOR CONTRIBUTIONS

Designed research and analyzed data, P.‐YD, EB, SD, JDM, XL, and F.‐XM; Enrollment and clinical management of study participants GE, FN, DR, and F.‐XM; Performed experiments, CB, MD, BT, J.‐MP, EF, and P.‐YD; Primary CML samples, conditioning, delivery, AB, SD; Original Draft P.‐YD and EB; Comments on the research direction and edition of the manuscript F.‐XM; Writing – Review and Edition, P.‐YD, F.‐XM, EB; Made Figures, P.‐YD and EB; Funding, P.‐YD, EF, and N.M
