# Supplementary material for: Killer immunoglobulin‐like receptor genotypes and chronic myeloid leukemia outcomes after imatinib cessation for treatment‐free remission
Source: Cancer Med. 2019 Jul 9;8(11):4976–85. doi: 10.1002/cam4.2371 (PMC6718597; doi:10.1002/cam4.2371)
Supplement: Supplementary file 1 [file CAM4-8-4976-s001.docx]

**Supplementary Table S1: *KIR* positive genotyping depending on recurrence status of 240 patients**

|  | **No relapse-*N* (%)**  ***N*=112 (46.7)** | | **Relapse-*N* (%)**  ***N*=128 (53.3)** | | ***P*-value** |
| --- | --- | --- | --- | --- | --- |
| ***KIR2DL1*** | 107 | (46.3) | 124 | (53.7) | 0.74 |
| ***KIR2DL2*** | 62 | (51.2) | 59 | (48.8) | 0.13 |
| ***KIR2DL3*** | 99 | (45.4) | 119 | (54.6) | 0.22 |
| ***KIR2DL4*** | 112 | (46.7) | 128 | (53.3) | - |
| ***KIR2DL5***  ***KIR2DL5A***  ***KIR2DL5B*** | 59  42  32 | (48.8)  (46.2)  (53.3) | 62  49  28 | (51.2)  (53.8)  (46.7) | 0.51  0.90  0.23 |
| ***KIR3DL1*** | 100 | (45.5) | 120 | (54.5) | 0.21 |
| ***KIR3DL2*** | 112 | (46.7) | 128 | (53.3) | - |
| ***KIR3DL3*** | 112 | (46.7) | 128 | (53.3) | - |
| ***KIR2DS1*** | 42 | (46.2) | 49 | (53.8) | 0.90 |
| ***KIR2DS2*** | 61 | (50.4) | 60 | (49.6) | 0.24 |
| ***KIR2DS3*** | 36 | (54.5) | 30 | (45.5) | 0.13 |
| ***KIR2DS4*** | 101 | (45.5) | 121 | (54.5) | 0.20 |
| ***KIR2DS5*** | 37 | (48.1) | 40 | (51.9) | 0.77 |
| ***KIR3DS1*** | 47 | (48.5) | 50 | (51.5) | 0.65 |

KIR: Killer Immunoglobulin-like Receptors

**Supplementary Table S2: Cox model for factors associated with time to 1^st^ DMR among 240 patients**

|  |  | **Univariate analysis** | | | **Multivariate analysis** | | |
| --- | --- | --- | --- | --- | --- | --- | --- |
|  | ***N*** | **HR** | **^95%^CI** | ***P*-value** | **aHR** | **^95%^CI** | ***P*-value** |
| **Age > 51 years†** | 180 | 0.82 | 0.61-1.11 | 0.20 | - | - | - |
| **Gender female** | 126 | 1.22 | 0.95-1.58 | 0.12 | 1.25 | 0.97-1.62 | 0.09 |
| **Sokal‡**  Intermediate  High | 100  32 | 1.06  0.81 | 0.80-1.39  0.54-1.20 | 0.69  0.29 | 0.77§ | 0.53-1.13 | 0.18 |
| ***KIR2DL1*** | 231 | 1.52 | 0.78-2.98 | 0.22 | - | - | - |
| ***KIR2DL2*** | 121 | 1.05 | 0.81-1.36 | 0.72 | - | - | - |
| ***KIR2DL3*** | 218 | 1.33 | 0.86-2.06 | 0.21 | - | - | - |
| ***KIR2DL4*** | 240 | - | - | - | - | - | - |
| ***KIR2DL5¶*** | 121 | 0.92 | 0.71-1.19 | 0.53 | - | - | - |
| ***KIR2DL5A*** | 91 | 0.88 | 0.68-1.15 | 0.37 | - | - | - |
| ***KIR2DL5B*** | 60 | 1.17 | 0.87-1.57 | 0.30 | 1.21 | 0.90-1.63 | 0.21 |
| ***KIR3DL1*** | 220 | 0.82 | 0.52-1.30 | 0.41 | - | - | - |
| ***KIR3DL2*** | 240 | - | - | - | - | - | - |
| ***KIR3DL3*** | 240 | - | - | - | - | - | - |
| ***KIR2DS1*** | 91 | 0.87 | 0.67-1.14 | 0.33 | - | - | - |
| ***KIR2DS2*** | 121 | 1.05 | 0.81-1.35 | 0.71 | - | - | - |
| ***KIR2DS3*** | 66 | 1.03 | 0.78-1.38 | 0.81 | - | - | - |
| ***KIR2DS4*** | 222 | 0.88 | 0.54-1.42 | 0.60 | - | - | - |
| ***KIR2DS5*** | 77 | 0.96 | 0.73-1.27 | 0.79 | - | - | - |
| ***KIR3DS1*** | 97 | 0.84 | 0.65-1.10 | 0.20 | - | - | - |

DMR: deep molecular remission; HR: hazard ratio; aHR: adjusted hazard ratio; CI: confidence interval;

† First quartile;

‡ Reference is low Sokal score (*N*=107);

§ Low and intermediate Sokal score have been associated for multivariate analysis (*N*=207);

¶ Reference is KIR2DL5A- B- (*N*=119).

**Supplementary Table S3: Cox model for factors associated with TFR among 240 patients**

|  |  | **Univariate analysis** | | | **Multivariate analysis** | | |
| --- | --- | --- | --- | --- | --- | --- | --- |
|  | ***N*** | **HR** | **^95%^CI** | ***P*-value** | **HR** | **^95%^CI** | ***P*-value** |
| **Age > 70 years†** | 60 | 1.18 | 0.80-1.74 | 0.40 | - | - | - |
| **Gender female** | 126 | 0.86 | 0.61-1.22 | 0.40 | - | - | - |
| **Sokal‡**  Intermediate  High | 100  32 | 1.01  1.56 | 0.69-1.47  0.95-2.56 | 0.97  0.08 | -  - | -  - | -  - |
| **Imatinib duration > 6 years§** | 120 | 0.66 | 0.46-0.93 | 0.02 | - | - | - |
| **Time to 1^st^ DMR ≥ 26 months§** | 120 | 0.77 | 0.54-1.09 | 0.14 | - | - | - |
| **DMR duration (years)¶**  > 4.4 | 60 | 0.58 | 0.37-0.91 | 0.02 | 0.58 | 0.37-0.91 | 0.02 |
| ***KIR2DL1*** | 231 | 1.36 | 0.50-3.67 | 0.55 | - | - | - |
| ***KIR2DL2*** | 121 | 0.79 | 0.56-1.12 | 0.19 | - | - | - |
| ***KIR2DL3*** | 218 | 1.42 | 0.72-2.79 | 0.31 | - | - | - |
| ***KIR2DL4*** | 240 | - | - | - | - | - | - |
| ***KIR2DL5*** | 121 | 0.93 | 0.66-1.31 | 0.66 | - | - | - |
| ***KIR2DL5A*** | 91 | 1.04 | 0.73-1.48 | 0.85 | - | - | - |
| ***KIR2DL5B*** | 60 | 0.82 | 0.54-1.24 | 0.36 | 0.84 | 0.55-1.27 | 0.40 |
| ***KIR3DL1*** | 220 | 1.50 | 0.73-3.06 | 0.27 | - | - | - |
| ***KIR3DL2*** | 240 | - | - | - | - | - | - |
| ***KIR3DL3*** | 240 | - | - | - | - | - | - |
| ***KIR2DS1*** | 91 | 1.01 | 0.71-1.44 | 0.96 | - | - | - |
| ***KIR2DS2*** | 121 | 0.84 | 0.60-1.19 | 0.33 | - | - | - |
| ***KIR2DS3*** | 66 | 0.76 | 0.51-1.14 | 0.19 | - | - | - |
| ***KIR2DS4*** | 222 | 1.49 | 0.69-3.19 | 0.31 | - | - | - |
| ***KIR2DS5*** | 77 | 1.00 | 0.69-1.44 | 0.99 | - | - | - |
| ***KIR3DS1*** | 97 | 0.95 | 0.67-1.36 | 0.79 | - | - | - |

TFR: treatment-free remission; DMR: deep molecular response; HR: Hazard ratio; CI: confidence interval;

† Fourth quartile;

‡ Reference is low Sokal (*N*=107);

§ Median;

¶ Reference is DMR duration 2.0-4.4 years (*N*=180).

**Supplementary Table S4: KIR *B*-content score and frequencies in each group**

| **KIR genotype** | ***B*-content score** | ***Cen*** | ***Tel*** | ***N* (%)** |
| --- | --- | --- | --- | --- |
| ***A/A*** | 0 | *A/A* | *A/A* | 78 (32.5) |
| ***B/x*** | 1 | *A/A* | *A/B* | 90 (37.5) |
|  |  | *A/B* | *A/A* |  |
|  | 2 | *A/A* | *B/B* | 49 (20.4) |
|  |  | *A/B* | *A/B* |  |
|  |  | *B/B* | *A/A* |  |
|  | 3 | *A/B* | *B/B* | 17 (7.1) |
|  |  | *B/B* | *A/B* |  |
|  | 4 | *B/B* | *B/B* | 6 (2.5) |


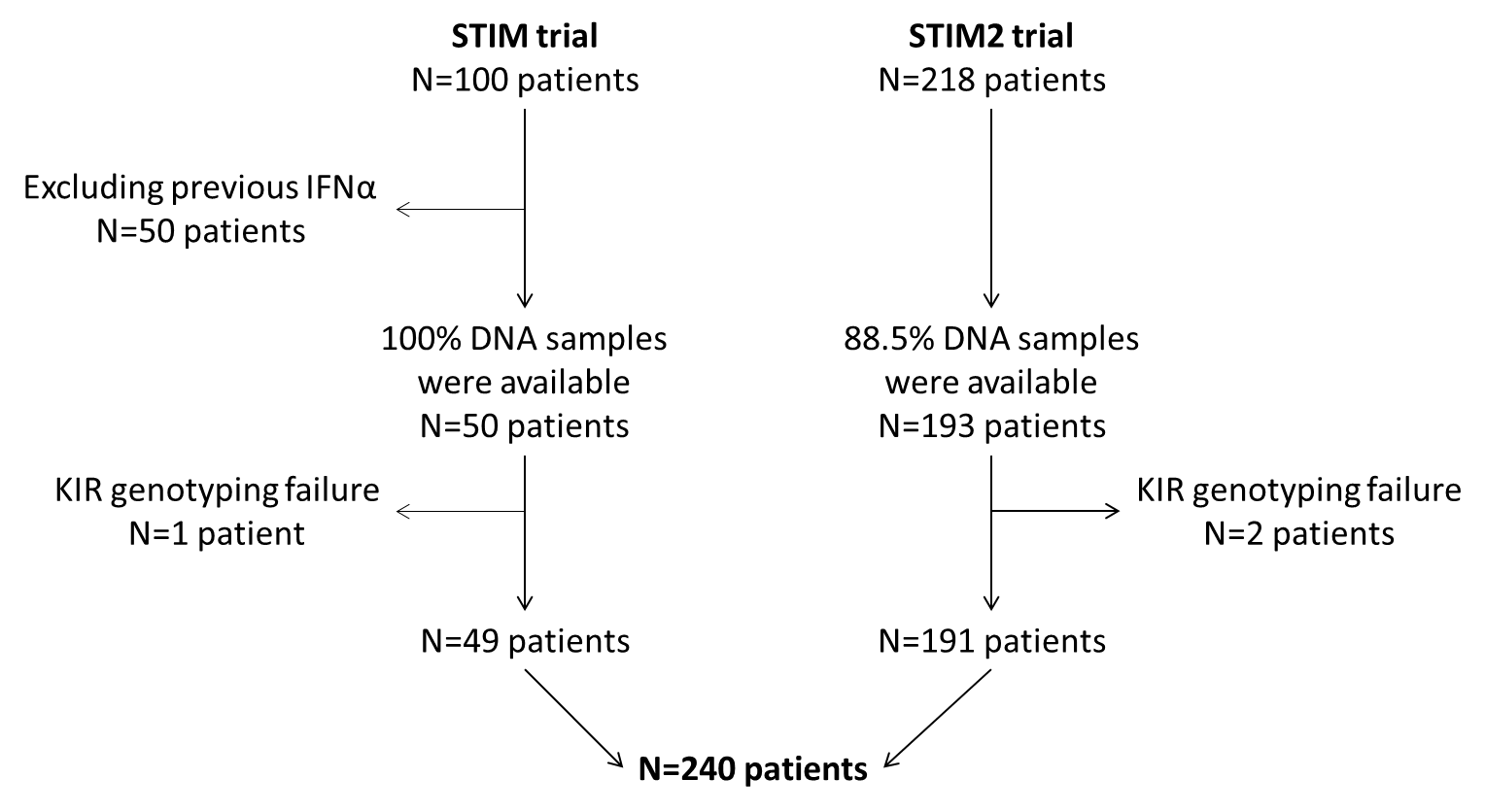


**Supplementary Figure S1: Study flow-chart**

**Supplementary Figure S2: Natural killer cells at imatinib discontinuation according to *KIR2DL5B* genotype.** Scatter dot plots represent CD3^-^ CD56^+^ NK-cell count, CD3^-^ CD56^dim^ NK-cell count and CD3^-^ CD56^bright^ NK-cell count for each individual according to *KIR2DL5B* genotype (*N*=24), median value and interquartile range are shown. *P*-values were not significant (Mann-Whitney test).
